# Abnormal Operation Detection of Automated Orchard Irrigation System Actuators by Power Consumption Level

**DOI:** 10.3390/s25020331

**Published:** 2025-01-08

**Authors:** Shahriar Ahmed, Md Nasim Reza, Md Rejaul Karim, Hongbin Jin, Heetae Kim, Sun-Ok Chung

**Affiliations:** 1 Department of Agricultural Machinery Engineering, Graduate School, Chungnam National University, Daejeon 34134, Republic of Korea; shahriar@o.cnu.ac.kr (S.A.); reza5575@cnu.ac.kr (M.N.R.); mrkarim@o.cnu.ac.kr (M.R.K.); 2 Department of Smart Agricultural Systems, Graduate School, Chungnam National University, Daejeon 34134, Republic of Korea; jhb0117@o.cnu.ac.kr; 3 National Institute of Agricultural Sciences, Rural Development Administration, Jeonju 54875, Republic of Korea; htkim1025@korea.kr

**Keywords:** smart agriculture, anomaly detection, orchard irrigation, operating status, irrigation actuators, signal processing

## Abstract

Information and communication technology (ICT) components, especially actuators in automated irrigation systems, are essential for managing precise irrigation and optimal soil moisture, enhancing orchard growth and yield. However, actuator malfunctions can lead to inefficient irrigation, resulting in water imbalances that impact crop health and reduce productivity. The objective of this study was to develop a signal processing technique to detect potential malfunctions based on the power consumption level and operating status of actuators for an automated orchard irrigation system. A demonstration orchard with four apple trees was set up in a 3 m × 3 m soil test bench inside a greenhouse, divided into two sections to enable independent irrigation schedules and management. The irrigation system consisted of a single pump and two solenoid valves controlled by a Python-programmed microcontroller. The microcontroller managed the pump cycling ‘On’ and ‘Off’ states every 60 s and solenoid valves while storing and transmitting sensor data to a smartphone application for remote monitoring. Commercial current sensors measured actuator power consumption, enabling the identification of normal and abnormal operations by applying threshold values to distinguish activation and deactivation states. Analysis of power consumption, control commands, and operating states effectively detected actuator operations, confirming reliability in identifying pump and solenoid valve failures. For the second solenoid valve in channel 2, with 333 actual instances of normal operation and 60 actual instances of abnormal operation, the model accurately detected 316 normal and 58 abnormal instances. The proposed method achieved a mean average precision of 99.9% for detecting abnormal control operation of the pump and solenoid valve of channel 1 and a precision of 99.7% for the solenoid valve of channel 2. The proposed approach effectively detects actuator malfunctions, demonstrating the potential to enhance irrigation management and crop productivity. Future research will integrate advanced machine learning with signal processing to improve fault detection accuracy and evaluate the scalability and adaptability of the system for larger orchards and diverse agricultural applications.

## 1. Introduction

High-value crops, especially fruit trees, are highly susceptible to water stress. Water stress, also known as water deficit, occurs when there is an insufficient supply of soil moisture to support the optimal growth and health of trees or plants [1]. This condition arises due to either a lack of available soil water or a high atmospheric evaporative demand, leading to adverse physiological effects on trees or plants [2]. With global warming set to shift precipitation patterns, droughts are expected to become more frequent, exacerbating water stress in orchards [3]. However, managing irrigation effectively is not only about addressing water deficit but also mitigating the problem of water oversupply. Over-irrigation can lead to waterlogging, nutrient leaching, and increased salinity in soil, which adversely affect root health and overall crop productivity [4]. This dual challenge underscores the importance of precision irrigation in orchard management—ensuring the right amount of water is delivered to crops at the right time.

According to the Korean Statistical Information Service (KOSIS), the apple (*Malus* × *domestica* Borkh.), a domesticated tree and fruit of the Rosaceae family [5], is one of the most widely cultivated tree fruits in the Republic of Korea [6]. Approximately 6% of the total agricultural land is allocated to fruit tree cultivation [7]. Fruit quality is significantly influenced by environmental factors, such as high temperature, water stress, and drought conditions, which affect the flavor and appearance of fruits by enhancing secondary metabolism and directly impact the quantity and quality of orchard harvests through irrigation management [8,9,10,11,12]. In conditions of limited water availability or excessive water delivery, effective irrigation management could ensure sustainable water use while maintaining high-quality orchard fruit production.

Advancements in agricultural technology are reshaping irrigation practices to improve efficiency and sustainability. Drip irrigation, widely adopted for orchard cultivation both in Korea and worldwide, delivers water at low rates directly to plant roots, preserving water and nutrients while minimizing disease risk [13,14]. Its slow, precise water distribution makes it adaptable to different soil types and achieves about 90% irrigation efficiency, compared to 70% for sprinklers and 50% for surface irrigation [10]. Moreover, effective irrigation management now relies on advanced monitoring systems that collect and analyze timely crop information through methodologies such as field-based measurements, remote sensing, and seamless integration of sensory and control systems [13,15,16,17]. The use of sensing technology for automation in the agricultural sector has significantly increased in the current decade, particularly in crop management [18,19,20]. Together, these advancements play a pivotal role in modernizing irrigation practices and enhancing sustainability in orchard management.

Building on advancements in agricultural technology, the integration of the Internet of Things (IoT) and sensors has further enhanced efficient irrigation practices by enabling the detection, collection, storage, and transmission of data [21]. IoT is considered the best option for smart irrigation management systems, as they, combined with user-friendly mobile applications, facilitate real-time monitoring and adjustment of irrigation schedules to suit environmental conditions [22,23]. However, challenges such as the need for custom software engineering to handle diverse sensors and actuators, as well as integration with mobile platforms, must be addressed for wider adoption [24,25]. Combining accurate, current sensing for monitoring the power consumption levels of irrigation actuators with IoT and smartphone applications can further enhance irrigation system efficiency. This approach could enable real-time malfunction detection and precise system adjustments.

Actuators, essential components in irrigation systems, vary depending on the type of agriculture and typically include pumps, valves, and sprinklers [26]. The solenoid valve, a critical element in fluid control systems, significantly impacts irrigation efficiency but is prone to malfunctions such as coil damage, contamination, mechanical wear, and improper installation [27,28]. Excessive heat can degrade coil insulation, preventing the valve from receiving electrical currents and causing operational failure [28]. Fault and anomaly detection mechanisms are vital for identifying issues like leaks, blockages, and malfunctioning valves early, ensuring optimal water delivery and resource conservation [29]. By implementing precise diagnostic systems, these faults can be detected and addressed promptly, reducing downtime, maintaining system efficiency, and supporting sustainable agricultural practices [30]. Early detection and resolution of actuator issues not only can enhance irrigation reliability but also extend the lifespan of the system, minimizing costs and environmental impact.

Detecting and regulating electric currents is vital in many electrical systems, including irrigation systems, as they help identify faults such as short circuits and open circuits. While different sensors are available, no single type is optimal for all applications [31]. Magnetic sensors, including fluxgate, the Hall effect, and magnetoresistive technologies, are effective due to their high isolation, compact size, and non-intrusive design [32]. Hall sensors, in particular, excel in measuring current or voltage variations, providing critical data for diagnosing faults by detecting the magnetic field produced by a current passing through a conductor [33,34]. Signal processing is equally vital to ensure data accuracy and mitigate interference from noise, enabling precise fault detection. High-quality signals are crucial for diagnosing faults effectively and ensuring irrigation systems operate efficiently in dynamic and unpredictable environments [35,36,37]. Fundamentally, accurate current sensing and reliable signal processing are essential for detecting faults in irrigation actuators, enhancing overall system performance.

Despite promising results in controlled environments, real-world applications face challenges due to dynamic conditions and the limited processing power of microcontrollers usually used in open-field irrigation systems, requiring scalable solutions for effective fault detection and system performance enhancement. Given these limitations, there is a clear need for more robust and efficient methods for detecting malfunctions in irrigation actuators, particularly in on-field settings where conditions are less controlled and sensor networks must operate reliably over extended periods. This study addressed this gap by developing a signal processing-based technique for detecting malfunctions in irrigation actuators. Unlike machine learning, signal processing offers a lightweight solution ideal for integration with microcontroller-based systems in automated irrigation. The objective of this study was to develop a method to detect abnormal operation status based on power consumption levels for automated orchard irrigation actuators.

## 2. Materials and Methods

### 2.1. Experimental Site, Sensors, and Irrigation Components

**Figure 1:**
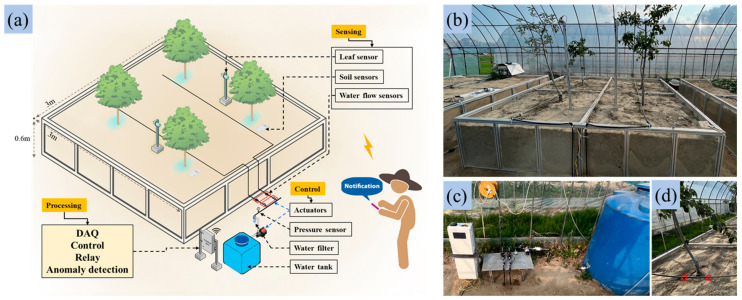
Experimental setup: (**a**) schematic diagram of the demonstration orchard with the irrigation system setup, (**b**) the test bench with soil, (**c**) drip irrigation system, and (**d**) watering area and emitter positions.

A demonstration apple orchard was established inside a plastic greenhouse at Chungnam National University, Daejeon, Republic of Korea (36°22′7.8″ N, 127°21′15.3″ E) to support the development and testing of an advanced irrigation monitoring system. The test bench size was 3 m × 3 m, featuring a square-shaped soil bed with a height of 0.6 m, enclosed by a hard plastic glass and aluminum profile structure, as shown in Figure 1a. The soil used in the demonstration orchard was sandy loam, composed of 78% sand, 14% silt, and 8% clay. Figure 1b illustrates the schematic diagram of the demonstration orchard and the layout of the irrigation system components. The demonstration orchard consisted of four apple trees arranged in two rows, with two trees per row. The average height and canopy area of the trees were 2 m and 1.1 m, respectively. The experiment was started on 17th July, running from 14:08 p.m. to 16:31 p.m., during which the irrigation system was actively monitored and data were collected.

A drip irrigation system was implemented on the test bench, with water supplied from a water tank using a 230-volt AC water pump (Model: 8090-212-246; Shurflo LLC., Costa Mesa, CA, USA) and two 24V AC solenoid valves (Model: S390T-2-R Bermad CS Ltd., Evron, Israel), as shown in Figure 1c. A pressure gauge and a pump controller (Model: BL01535; H Plus Mall Co., Ltd., Shanghai, China) were used to regulate water pressure and discharge rates to prevent overpressure. The system included two rows with four emitters, spaced at 0.2 m intervals along rows separated by 1.4 m, delivering water at 4 L/h through a 12.5 mm diameter dripline. Flow rates were monitored using two water flow sensors, and the watering area was defined as 0.4 m on each side of the tree rows to ensure precise irrigation for apple trees, considering the smaller canopy area and height of the trees used in the study [10]. Figure 1d illustrates the layout, and Table 1 outlines the specifications of the actuators used in this study.

**Figure 2:**
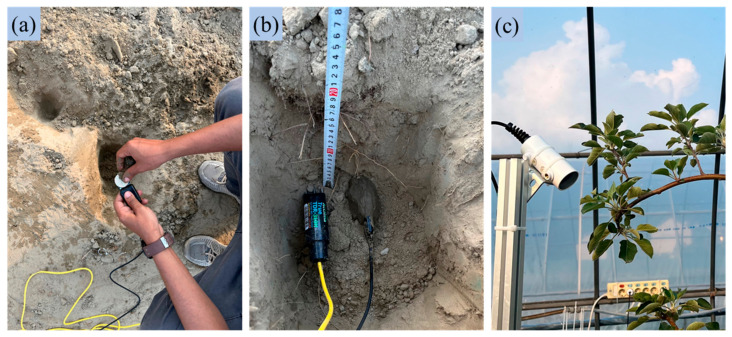
Installation of sensors in the demonstration orchard: (**a**) wet soil addition for soil water potential measurement, (**b**) placement of soil water content and water potential sensors, and (**c**) placement of leaf temperature sensor.

An effective irrigation strategy maintains soil moisture levels between field capacity and the threshold of water stress, ensuring a continuous supply of water for the crops. Although apple tree roots can extend several meters into the ground, the majority of mature roots are concentrated within the upper 75 to 90 cm of soil [10]. In this study, soil water content and potential were monitored using the True TDR-310H (Acclima, Inc., Meridian, ID, USA) and the TEROS 21 (Meter Group, Inc., Pullman, WA, USA) sensors, installed at a depth of 25 cm following the methodology of [38], as shown in Figure 2a,b. Each orchard was equipped with a set of these sensors for real-time monitoring of the soil water content and potential. Leaf temperature was monitored using SI-431-SS infrared radiometers (Apogee Instruments, Inc., North Logan, UT, USA), positioned at an angle in each row to effectively capture apple tree leaf temperatures, as shown in Figure 2c [39].

**Figure 3:**
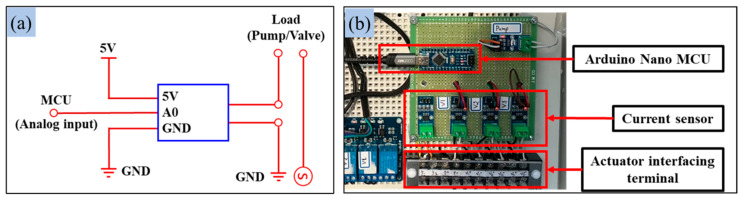
Sensor interfacing for measuring actuator power consumption using current sensors: (**a**) circuit diagram, and (**b**) sensors installation with the microcontroller.

Measuring signals using current sensors provides valuable insights into the power consumption behavior of the pump and solenoid valves. In this study, current sensors (ACS-712-05B and ACS-712-20A, Allegro MicroSystems, Manchester, NH, USA) were used to measure the power consumption of the irrigation system’s pump and solenoid valves. Table 2 provides detailed specifications for these sensors. Each actuator was connected to a sensor at the load terminal, with the analog output data fed to a primary microcontroller (Model: Arduino Nano, Arduino, Monza, Italy), as shown in Figure 3. The microcontroller converted the Hall effect-based analog voltage outputs into the root-mean-square (RMS) current values using Equation (1) [40,41]:

(1) $$I=\frac{\left(V_{out}-V_{zero}\right)}{Sensitivity}$$

where I is the current, V_out_ is the sensor output voltage, V_zero_ is the zero-current voltage (2.5 V for ACS712), and sensitivity is the sensor’s mV/A rating.

The sensors, with sensitivities of 185 mV/A (ACS712-05B) and 100 mV/A (ACS712-20A), were calibrated for accuracy. AC current was measured using Equation (2) [33]:

(2) $$I_{RMS}=\sqrt{\frac{\sum I_{now}^{2}}{Measurements\;Count}}$$

where I_RMS_ is the RMS current, and I_now_ represents the instantaneous current measurement.

### 2.2. Irrigation Control and Data Acquisition

**Figure 4:**
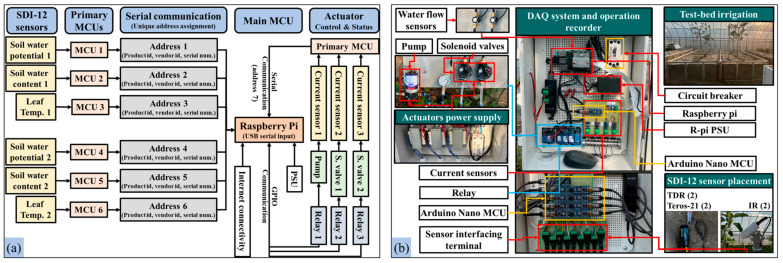
Irrigation control and data acquisition system: (**a**) schematic diagram of the sensor interfacing with the primary and secondary MCUs, and (**b**) data acquisition flow from the demonstration orchard.

The irrigation actuators were controlled using 5 V relay modules (Model: SRD-05VDC-SL-C, Songle, Yuyao, China) managed by a secondary microcontroller (Model: Raspberry Pi 4B, Raspberry Pi Foundation, Cambridge, UK). A time-based program was developed to automate the actuator operation at 60-s intervals over a four-hour experimental period. Each soil and leaf sensor was connected to a primary microcontroller (MCU) through GPIO pins, as shown in Figure 4. Sensor data acquisition was acquired at 10-s intervals using C++ instructions uploaded to the primary MCUs. These MCUs, responsible for soil and leaf sensor data and actuator power measurements, communicated with the secondary MCU via USB serial ports. Table 3 outlines the specifications of both MCUs used in this study.

Each primary MCU was assigned a unique address to facilitate efficient USB serial data transfer, managed with Python-based code. The used soil and leaf sensors (SDI-12 protocol-based) generated commas-separated data streams, which the secondary MCU converted to ASCII strings for readability. Parsed data included soil water content, potential, and leaf temperature.

The irrigation control and data collection were automated using a Python (v3.13) program on the secondary MCU, with results displayed on a touch panel for easy observation. Control commands for the actuators were recorded as binary data (‘1’ for ‘On’ and ‘0’ for ‘Off’) to monitor their operational states. Python multi-threading ensured smooth, concurrent data acquisition, control, and monitoring preventing delays or data loss.

**Figure 5:**
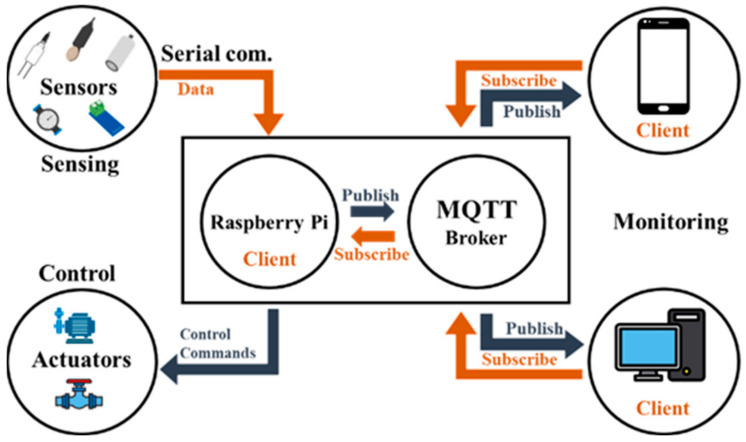
Schematic diagram of MQTT communication protocol between the irrigation systems and smartphone application.

A smartphone application was developed using Java (v17.0.10) and the Android SDK (v35.0.1) [25] to enable real-time remote monitoring and control of the irrigation system. Message Queuing Telemetry Transport (MQTT), a lightweight protocol commonly used on low-powered devices, facilitated machine-to-machine (M2M) communication between the secondary MCU and the smartphone application [42,43]. An MQTT broker was installed on the secondary MCU that transmitted all the data using the ’publish’ method to the smartphone (Figure 5) [44]. Irrigation was triggered by pressing a designated button in the smartphone application, which sent a control command via the ’subscribe’ method to the secondary MCU. Sensor readings and actuator states were stored locally on the smartphone in a Comma-Separated Values (CSV) file to ensure data integrity and support future analysis.

### 2.3. Data Analysis and Anomaly Detection

**Figure 6:**
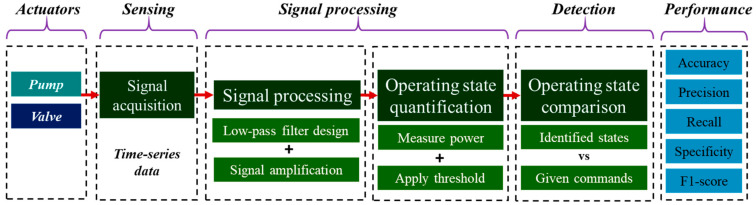
Overall, signal processing and anomaly detection workflow were used in this study.

This study introduces a real-time, lightweight threshold-based anomaly detection method tailored for irrigation actuators. Unlike existing approaches that rely on complex machine learning models, this method leverages signal processing to provide resource-efficient and scalable detection suitable for automated orchard systems. Figure 6 illustrates the schematic diagram of the process of time-series signal acquisition and signal processing steps for detecting normal or abnormal control operation of the actuators for the irrigation system. The first step in this method involves collecting time-series raw data from the current sensors connected to the actuators. These data are then subjected to real-time signal processing to accurately capture and prepare the power consumption data for analysis. The signal processing steps include applying a real-time low-pass filter to remove noise, amplifying the signal, and deducting offset values to ensure a precise representation of the power consumption behavior of actuators. After processing, the measured power values are evaluated using predefined threshold values to identify the ‘On’ and ‘Off’ states of the actuators. By comparing the control commands with the power consumption patterns, the system can detect any deviations and identify abnormal operations.

#### 2.3.1. Signal Processing to Determine Power Consumption Level

The raw signals from sensors often show sudden shifts in amplitude between consecutive samples, which can negatively impact the performance of the application [45]. To address this issue, a real-time first-order low-pass filter was designed and applied to the input signals from the current sensors within the primary MCU before the experiment began. The first-order low-pass filter was implemented, with the continuous-time transfer function defined as Equation (3) [46,47], as follows:

(3) $$H(s)=\frac{\omega_{0}}{s+\omega_{0}}$$

where H(s) is the transfer function, $\omega_{0}$ is the cut-off frequency, and s is the complex frequency variable.

However, the digital implementation of filters operates in discrete time. Tustin’s method (bilinear transform) was applied [48] to discretize the transfer function. In this method, the continuous-time variable s was replaced using Equation (4) as follows:

(4) $$s=\frac{2}{∆t}(\frac{1-z^{-1}}{1+z^{-1}})$$

where ∆t is the sampling period, and z^−1^ represents the discrete-time shift operator. Substituting this into the continuous-time transfer function, the discrete-time transfer function was obtained via Equation (5):

(5) $$H(z)=\frac{\omega_{0}}{\frac{2}{∆t}(\frac{1-z^{-1}}{1+z^{-1}})+\omega_{0}}$$

This simplifies to

(6) $$H(z)=\frac{∆t\omega_{0}+({∆t\omega_{0})z}^{-1}}{(∆t\omega_{0}+2)+(∆t\omega_{0}-2)z^{-1}}$$

where the coefficients are defined as

$$b_{0}=b_{1}=\frac{\alpha }{\alpha +2}$$

$$a_{1}=-\frac{\alpha -2}{\alpha +2}$$

For the discrete filter update, the output y [n] was calculated as:

(7) $$y\left[n\right]=a_{1}y\left[n-1\right]+b_{0}x\left[n\right]+b_{1}x[n-1]$$

These coefficients were derived directly from the discrete transfer function, expressed as

(8) $$H(z)=\frac{b_{0}+b_{1}z^{-1}}{1-a_{1}z^{-1}}$$

By setting α = ω_0_. ∆t, the transfer function becomes

(9) $$H(z)=\frac{\alpha +\alpha z^{-1}}{(\alpha +2)+(\alpha -2)z^{-1}}$$

This can be rewritten as

$$H(z)=\frac{\frac{\alpha }{\alpha +2}+\frac{\alpha }{\alpha +2}z^{-1}}{1+\frac{\alpha -2}{\alpha +2}z^{-1}}$$

The low-pass filter coefficients were calculated by defining the sampling period (dt) as one millisecond, corresponding to a sampling frequency of 1 kHz. The cut-off angular frequency ($\omega_{0}$) was set to 5 Hz, which was converted to radians per second by multiplying this by 2π. Using the relationship α = $\omega_{0}$ × dt, the intermediate parameter α was calculated, which played a crucial role in determining the filter coefficients. The filter coefficients were then computed using specific formulas: the feedback coefficient a [0] was determined as a [0] = (α − 2)/(α + 2), and the feedforward coefficients b [0] and b [1] were both calculated as b [0] = b [1] = α/(α + 2). For the specified sampling period and cut-off frequency, these coefficients were computed in the Arduino environment, yielding the following values:

*b*_0_ = *b*_1_ = 0.015465039003346996

*a*_1_ = 0.9690699219933061

**Figure 7:**
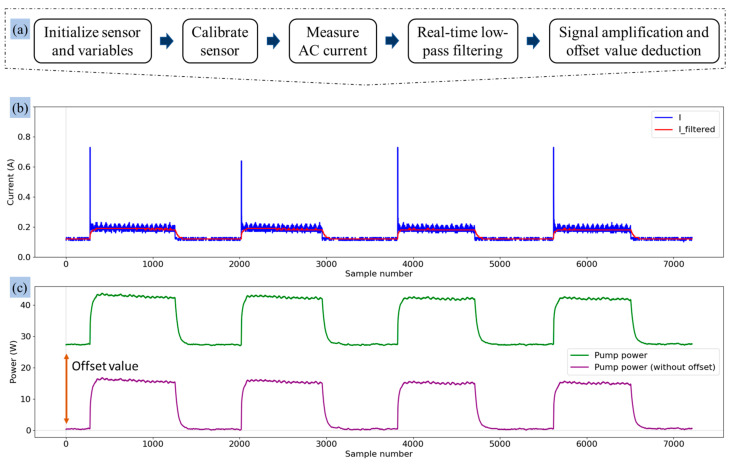
Real-time signal processing for estimating power consumption: (**a**) flowchart of the signal processing and power calculation, (**b**) low-pass filter application, and (**c**) signal amplification and offset value deduction.

After determining the coefficients, the following equation was implemented on the secondary MCU to facilitate real-time low-pass filtering:

(10) $$I_{filtered}=0.969\times I_{yn1}+0.0155\times I+0.0155\times I_{xn1}$$

where I_filtered_ is the processed signal, I_yn1_ is the previous output value of the filter (from the last time step), I is the raw signal (the current value being measured at the current time step), and I_xn1_ is the previous input value of the current (from the last time step). Figure 7 illustrates the schematic flowcharts for estimating signal processing and power consumption, illustrating the sequential phases involved in both processes. Figure 7b illustrates the conversion of the raw signal to the filtered signal for a water pump under laboratory conditions, where the pump was turned ‘On’ four times and turned ‘Off’ four times with the help of a relay module. The low-pass filter effectively eliminated the sudden peaks created when turning on the pump and reduced noise from the raw signal. Figure 7c represents the signal amplification and offset value deduction, which created a precise pattern to distinguish between the ‘On’ and ‘Off’ states of the pump during the test. The instantaneous power consumption was calculated using Ohm’s law by multiplying the measured current by the known supply voltage. The formula used for power calculation or amplification was

(11) $$P=U\times I_{RMS}$$

where P is the power (W), U is the supply voltage (230 V or 24 V), and I_RMS_ is the measured current (A).

#### 2.3.2. Actuator Control State Classification and Abnormal Operation Detection

Extracting statistical features from current sensor inputs is one of the simplest methods for time-domain feature extraction [49]. The histogram is a widely used graphical tool in statistical analysis that represents data distributions by showing the frequency of value ranges in time series or other datasets. It plays a crucial role in applications such as shape matching, image retrieval, enhancement, normalization, feature matching, and visual tracking [50,51]. To identify the control states, the general behavior of actuators was tested under laboratory conditions, free from environmental influences, prior to the irrigation experiment in the greenhouse. Each actuator was individually connected to a current sensor, and measurements were taken using the real-time low-pass filter. Power consumption data were recorded at a frequency of 50 Hz, consistent with the characteristics of the AC signal, and stored in a CSV file. Alongside raw and filtered signal data, control commands were logged in a separate column to indicate the operational state of the actuator, with ‘1’ representing ‘On’ and ‘0’ representing ‘Off’. Any offset values in the power consumption data were adjusted to account for baseline noise or drift, ensuring measurement accuracy. The dataset was then divided into two distinct subsets: the ‘On’ state, comprising rows where the control command was ‘1’, and the ‘Off’ state, consisting of rows where the control command was ‘0’. This division allowed for a focused analysis of power consumption across both operational states. Two approaches were considered to classify the operational states of actuators based on power consumption: the minimum–maximum (Min–Max) power consumption range and a manual range approach [52,53]. Histograms of the power consumption of actuators data during the ‘On’ and ‘Off’ states were plotted, offering a visual representation of the power consumption distribution during this test. In the Min–Max approach, the lowest power consumption value from the ‘On’ state and the highest power consumption value from the ‘Off’ state were used as threshold values. In the manual approach, threshold values were selected manually based on the closest lowest point of the ‘On’ state cluster and the highest point of the ‘Off’ state cluster. These thresholds were carefully chosen to effectively distinguish between ‘On’ and ‘Off’ states, ensuring accurate classification of the operation of the actuators. A confusion matrix was generated to evaluate the accuracy of the classification for both methods to effectively distinguish between the ‘On’ and ‘Off’ states.

**Figure 8:**
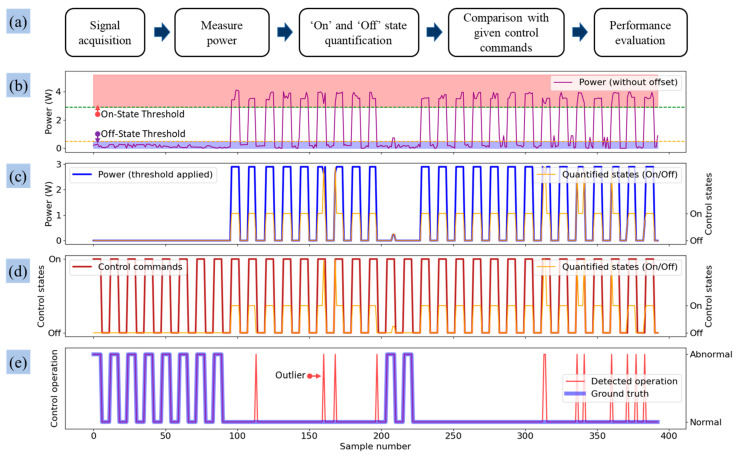
Detailed processing steps for detecting abnormal operation: (**a**) schematic diagram of abnormal operation detection, (**b**) visualization of threshold values on measured power, (**c**) classification of operating states after applying threshold values, (**d**) comparison of classified states with given control commands, and (**e**) performance evaluation by comparing detected operation with ground truth.

A Python-based algorithm was developed to detect abnormal operations of the irrigation actuators. The overall methodology for detecting abnormal operations is presented in Figure 8a. Initially, threshold values determined from lab tests were applied to the measured time-series power data of the actuators, as illustrated in Figure 8b. Following the application of these thresholds, the signals were classified into binary values of ‘1’ and ‘0’, representing the operational states ‘On’ and ‘Off’, respectively (Figure 8c). Subsequently, these states were compared with the actual control commands recorded during the tests, which were also represented as binary values, as shown in Figure 8d.

The process of identifying ‘abnormal’ operations from the power consumption data involves classifying actuator control actions as either ‘normal’ or ‘abnormal’. An operation is labeled as ‘abnormal’ if the control status, as determined from power consumption data, does not correspond to the given control command. Conversely, the operation is classified as ‘normal’ when the control status aligns with the given command. In the final step, the detected operations were compared with the ground truth values to evaluate the performance of the developed algorithm, as illustrated in Figure 8e. These ground truth values were determined through both visual observation and expert judgment. To assess the algorithm’s performance, 393 data sets were analyzed to identify any instances of ‘abnormal’ operations during the irrigation control process of the time-based irrigation system.

Unlike machine learning models, which typically require training and testing phases, this study employed a threshold-based detection approach, thereby eliminating the need for such phases. The performance of the detection method was evaluated using standard metrics, including precision, recall, F1 score, and accuracy, as defined in Equations (12)–(15) [54,55]:

(12) $$Precision=\frac{TP}{TP+FP}$$

(13) $$Recal=\frac{TP}{TP+FN}$$

(14) $$F1score=\frac{2\times Precision\times Recall}{Precision+Recall}$$

(15) $$Accuracy=\frac{TP+TN}{TP+FN+FP+TN}$$

where TP represents true positive detections, FN represents false negative detections, FP represents false-positive detections, and TN represents true negative detections. Additionally, the mean average precision (mAP) was calculated as the average precision across different classes, as shown in Equation (16) [56]:

(16) $$mAP=\frac{\sum_{i=1}^{m}AP_{i}}{m}$$

## 3. Results

### 3.1. Irrigation Control Status and Sensor Data Monitoring

**Figure 9:**
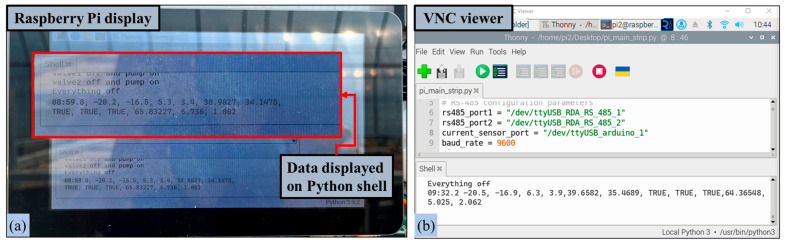
Onsite monitoring of the irrigation system using secondary MCU: (**a**) monitoring in Raspberry Pi display and (**b**) monitoring in VNC viewer.

For onsite monitoring, the secondary MCU successfully collected data from all sensors distributed throughout the irrigation system, including those measuring soil water content, water potential, leaf temperature, and actuator power consumption. It also processed the control commands for the actuators. The successful outcome of this demonstration is shown in Figure 9a, which illustrates the real-time data displayed on the screen continuously and without any interruptions. Moreover, the capability to remotely access the secondary microcontroller via a virtual network computing (VNC) viewer [57] further enhances the system’s flexibility, allowing users to monitor the irrigation system from any location with internet access, as shown in Figure 9b.

**Figure 10:**
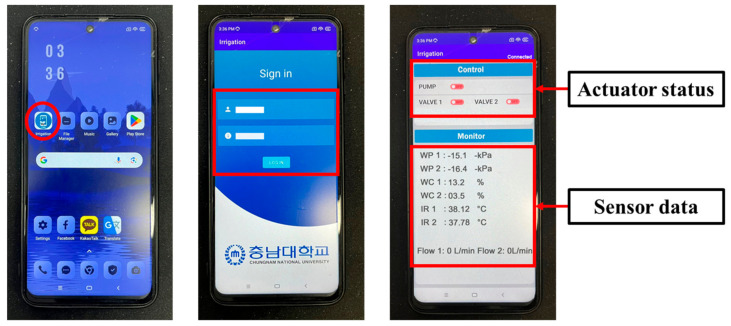
Remote monitoring of the irrigation system on the Android application.

After successfully logging into the application with the required credentials, it was observed that the transfer of sensor data from the secondary MCU to the Android application via MQTT was consistent. The monitoring section of the Android application provided a clear, real-time visual representation of the sensor data, and the control section of the application displayed the operational status of the actuators. These results demonstrate seamless system interoperability. Figure 10 illustrates the continuous monitoring of sensor data and actuator status during the study.

**Figure 11:**
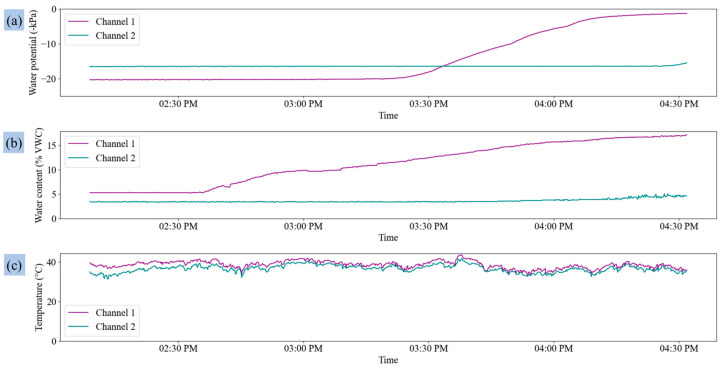
Variation in the sensor data after irrigation control: (**a**) soil water potential, (**b**) soil water content, and (**c**) leaf temperature.

The data from the CSV file saved in smartphone storage were visualized, as shown in Figure 11. The variation in soil water potential, water content, and leaf temperature after irrigation control is shown in Figure 11a–c. The soil water content sensor demonstrated a quicker response compared to the soil water potential sensor, reflecting changes in moisture levels almost immediately after irrigation. However, a slight delay was observed in the soil water content changes in the second channel compared to the first channel, likely due to malfunctioning of the valve in the second channel, which required more time for moisture absorption and detection. This delay highlights the sensitivity of the irrigation system to varying soil conditions between different irrigation zones.

### 3.2. Determining the Threshold Value for the Control Operation

**Figure 12:**
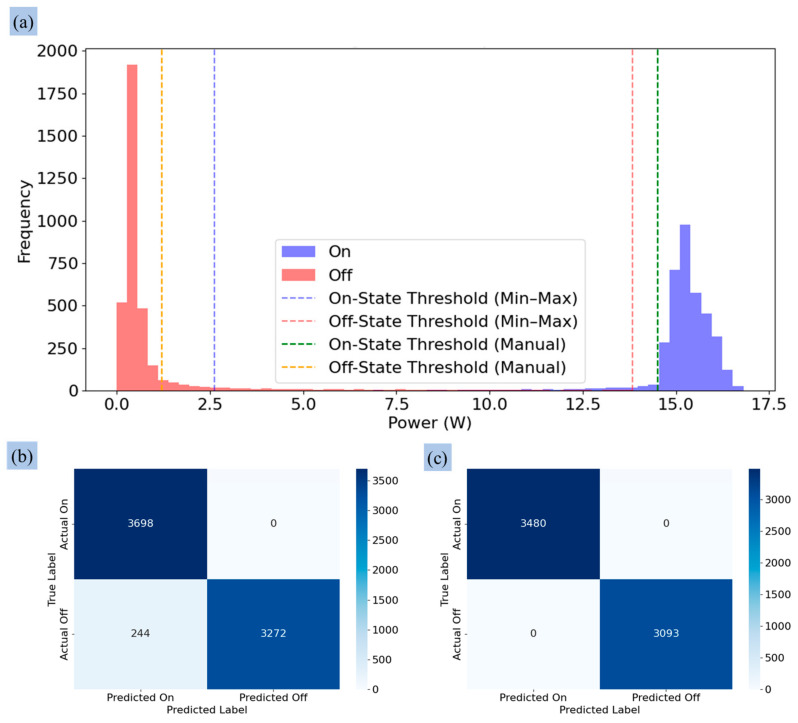
Histogram analysis of power consumption behavior of the pump: (**a**) threshold value application using Min–Max and manual approaches, (**b**) performance of Min–Max threshold approach, and (**c**) performance of manual threshold approaches.

**Figure 13:**
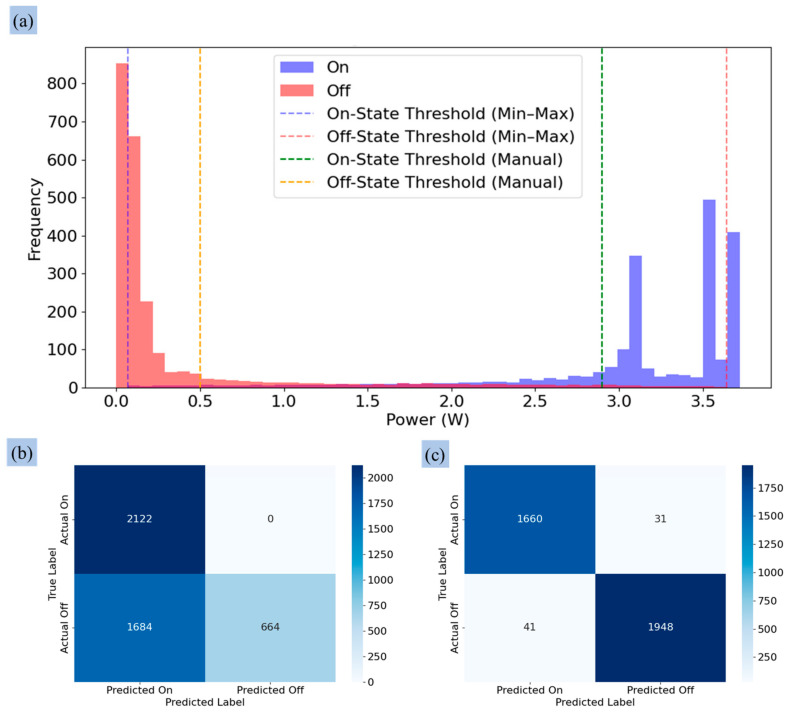
Histogram analysis of power consumption behavior of the valve: (**a**) threshold value application using Min–Max and manual approaches, (**b**) performance of Min–Max threshold approach, and (**c**) performance of manual threshold approaches.3.3. Detecting Abnormal Operation of the Actuators.

Figure 12 and Figure 13 provide a comprehensive analysis of the power consumption behavior of pumps and valves, respectively, using Min–Max and manual threshold approaches for classifying their operational states (‘On’ and ‘Off’). The histogram in Figure 12a illustrates the distribution of the pump under the Min–Max and manual threshold methods. The thresholds were set at a minimum of 2.62 W for the ‘On’ state and a maximum of 13.84 W for the ‘Off’ state. The histogram reveals a significant overlap in power values between these two states, resulting in an uncertain zone between the ‘On’ state minimum threshold (2.62 W) and the ‘Off’ state maximum threshold (13.84 W). This overlap created an uncertain zone, leading to misclassifications. The confusion matrix in Figure 12b shows the impact of this overlap, with 3698 true positives (TPs), 3272 true negatives (TNs), and 244 false negatives (FNs), though no false positives (FPs) were recorded.

In comparison, the manual threshold method, shown in the histogram in Figure 12a, used practically determined thresholds of 14.5 W for the ‘On’ state and 1.2 W for the ‘Off’ state based on observed clustering of power consumption data. This method minimized overlap, resulting in a clearer separation of two states and improved classification accuracy. As shown in Figure 12c, the manual method achieved 3480 TPs and 3093 TNs, with no FNs or FPs, providing a perfect classification of pump states.

Figure 13a presents the histogram analysis of the valve’s power consumption, comparing the Min–Max and manual threshold methods. For the Min–Max approach, thresholds were set at 0.068 W for the ‘On’ state and 3.64 W for the ‘Off’ state. Similar to the pump analysis, this approach showed substantial overlap between the states, resulting in classification errors. The confusion matrix in Figure 13b reveals 2122 TPs, 664 TNs, and 1684 FNs, with no FPs.

In contrast, the manual threshold approach involved manually setting the thresholds at 2.8 W for the ‘On’ state and 0.5 W for the ‘Off’ state based on the observed data clusters (Figure 13a). This approach significantly reduced overlap, as seen in Figure 13c, which reports 1660 TPs, 1948 TNs, 41 FNs, and 31 FPs. The reduced misclassification rates highlight the manual method’s superior performance in distinguishing valve states.

Overall, Figure 12 and Figure 13 highlight the limitations of the Min–Max threshold approach in differentiating operational states for the pump and valve due to overlapping power ranges. This comparative analysis highlights the manual approach as superior for pump and valve state classification, as it significantly reduces false positives and negatives. This enhanced accuracy ensures precise irrigation scheduling, minimizing classification errors while optimizing water usage and resource allocation—key factors for sustainable agricultural practices.

The test results, summarized in Table 4, provide a detailed assessment of the performance of the actuator operation detection system across a pump and two solenoid valves. For the pump, out of 387 instances of actual normal operation, the detection model accurately identified 322 instances as normal. However, it failed to detect 6 abnormal instances and identified 64 outliers. These outliers suggest that the system occasionally misclassified normal operations, pointing to potential issues with the sensitivity of the threshold settings or the detection algorithm in distinguishing borderline cases between normal and abnormal behavior. The system demonstrated higher accuracy for the first solenoid valve, with 387 instances of actual normal operation. It correctly identified 359 operations as normal and detected no false abnormal operations. However, 21 outliers were recorded, indicating minor discrepancies in detection, though the system showed strong consistency in recognizing normal operations. These outliers may be attributable to slight variations in power consumption that were not significant enough to trigger an abnormal flag but still deviated from the expected normal range.

In the case of the second solenoid valve, which had 333 instances of actual normal operation and 60 instances of actual abnormal operation, the model demonstrated a balanced detection capability. It accurately classified 316 instances as normal and correctly identified 58 of the abnormal instances. Despite this, 14 outliers were observed, suggesting a misclassification or error in detecting abnormal operations. The model’s performance on the second solenoid valve highlights its capability to handle both normal and abnormal operational states effectively, though the presence of these outliers points to the need for potential refinement in distinguishing subtle anomalies.

Overall, the actuator operation detection system exhibited good performance in identifying normal operations, especially for the solenoid valves, where the detection accuracy was higher. However, the presence of outliers and occasional inconsistencies in identifying abnormal operations, particularly for the pump, suggest that the thresholds or detection algorithms could benefit from further optimization. Improving the model’s ability to consistently recognize abnormal states without increasing false positives will be key to enhancing its reliability and robustness.

**Figure 14:**
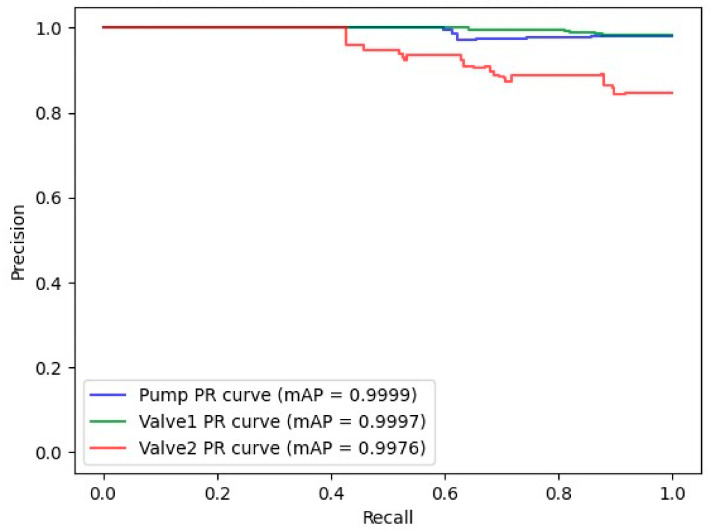
Precision–recall curve of the abnormal operation detection model.

The precision–recall (P-R) curve is an effective tool for assessing prediction performance in cases where the class imbalance is present. Figure 14 illustrates the P-R curve, showing that the proposed model achieved a high level of accuracy in detecting control operations. The model demonstrated strong proficiency in learning the relevant features and establishing a solid connection between the input data and control operations, as reflected by its low loss values and high mean average precision (mAP) scores. The mAP for the pump and solenoid valve of channel 1 was 99.99%, while for the solenoid valve of channel 2, it was 99.76%. These results indicate the model’s robust performance across all tested components.

Table 5 presents the anomaly detection results for the pump and solenoid valves across key performance metrics, representing the system’s accuracy and effectiveness in identifying both normal and abnormal operations. For the pump, the system achieved an accuracy of 99.70%, with a precision of 100% and a recall rate of 99.69%. The F1 score was 99.84%, indicating better performance in accurately identifying operational states with minimal errors. For the first solenoid valve, the system demonstrated an accuracy of 98.12%, a precision of 100%, and a recall rate of 98.09%. The F1 score was 98.91%, reflecting high accuracy in detecting normal operations, though there were minor discrepancies in the recall. The second solenoid valve exhibited an accuracy of 98.68%, with a precision of 100% and a recall rate of 98.44%, leading to an F1 score of 99.22%. The system achieved a specificity of 100% for all components, highlighting its robust capability to distinguish between normal and abnormal operations.

Overall, the detection system performed exceptionally well across all metrics, particularly in terms of precision and F1 score. While slight variations in recall rates suggest areas for improvement to enhance detection consistency, the system’s anomaly detection capabilities play a critical role in maintaining irrigation efficiency by identifying irregularities in actuator performance early. This helps prevent prolonged water loss, uneven irrigation, and potential damage to crops. Ensuring consistent soil moisture levels directly contributes to crop health and productivity while reducing system downtime minimizes maintenance costs—benefits that are particularly valuable for large-scale agricultural operations.

## 4. Discussion

The proposed system combines advanced threshold-based classification and anomaly detection with remote monitoring capabilities, delivering significant benefits to agricultural practices. Precise control of irrigation actuators ensures efficient water usage, reduces waste, and prevents system failures. These improvements lead to better crop yields and lower operational costs, particularly in water-scarce regions where optimized irrigation is vital.

This study demonstrated the effectiveness of an automated irrigation control system using a microcontroller unit (MCU) for both controlling irrigation actuators and monitoring sensor data remotely via a smartphone application. The system also successfully detected potential malfunctions in the irrigation actuators, contributing to improved reliability and maintenance. The Python-based control program, embedded in the secondary MCU, proved effective in receiving commands from the smartphone application and executing appropriate actions to manage the irrigation process. This capability aligns with findings from similar studies as described in [58], where sensor data were transmitted from the irrigation field to a smartphone application for remote irrigation control. The results from the CSV file confirm the functionality of the irrigation system, as the pump and valves were triggered to turn ‘On’ and ‘Off’ at 10-s intervals upon receiving the start command. Additionally, control commands were successfully stored as binary values, as intended. Such systems allow users to monitor and manage irrigation operations remotely, enhancing the efficiency and responsiveness of the system to environmental conditions.

The use of a smartphone application for irrigation scheduling for cotton cultivation by integrating soil moisture data from a smart sensor array was demonstrated by [25]. This contrasts with the present study, which highlights the potential for integrating a wider variety of sensors, such as those for monitoring soil water content, soil water potential, and leaf temperature, into a single smartphone-controlled system. Although the developed system used multiple sensors for measuring the soil water content, soil water potential, and leaf temperature, the irrigation control was based on a time-based schedule rather than real-time environmental data. This capability would allow for more responsive, data-driven irrigation management in different locations within smartphone applications.

Anomalies were detected during the irrigation process, specifically in the second valve, which affected the soil water level in the corresponding channel (Figure 11a,b). This demonstrated the system’s capability to identify and report abnormal operations, thereby preventing inefficient water use and system failures. Future studies could further enhance this functionality by incorporating cloud-based systems to manage larger datasets and improve decision-making efficiency through advanced data analytics.

This study successfully developed a smartphone application utilizing the MQTT protocol for efficient remote monitoring and control of an irrigation system. By integrating communication technologies like BLE and MQTT with hardware such as ESP32, Android devices, and Raspberry Pi, the system supports rapid deployment, similar to the smart city applications proposed in [59]. A similar study [58] showed the effectiveness of using Bluetooth connections to transfer sensor data to smartphone applications, where the data were stored efficiently in CSV format for easy access and further analysis. Building on this, the current study demonstrated the successful integration of a broad range of sensors utilizing the MQTT protocol, allowing for real-time data transmission from multiple MCUs to a centralized smartphone application. As described in [60], MQTT is well suited for environments requiring high concurrency, making it an ideal choice for systems that rely on real-time data transfer from multiple sensor nodes, as exemplified in the present study.

**Figure 15:**
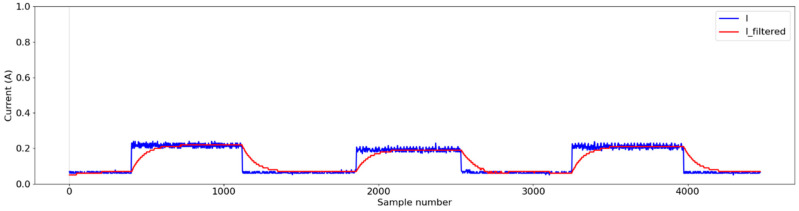
The low-pass filter was applied to the collected raw signal of the valve during lab conditions.

The signal processing results show the effectiveness of the low-pass filter designed to differentiate the operational states of actuators, as shown in Figure 12 and Figure 13. Signal smoothing, as described in [61], is essential for reducing distortions by adjusting sample amplitudes relative to neighboring samples. The discrete low-pass digital filter applied during laboratory tests effectively processed the signals from the pump by suppressing transient peaks upon activation without altering the fundamental characteristics of the signals (Figure 7b). This demonstrated the capacity of the filter to preserve key signal features while minimizing noise. However, the lower power consumption of the valves resulted in minimal peak generation during state transitions, and the filter occasionally over-smoothed the signal, as shown in Figure 15. Despite this, the 10-s sampling frequency mitigated any significant impact of over-smoothing during the greenhouse tests. Similar trends were observed in previous studies applying filters to raw signals [61,62,63].

The system operated for approximately two hours, but only one hour of data was used for power consumption analysis due to instability at the beginning of the experiment. The manual threshold approach improved classification accuracy for both pump and valve operations. For the pump, it completely eliminated false positives and negatives, achieving high classification performance. For the valve, the manual method minimized false classifications compared to the Min–Max method, accurately detecting valve states. The histograms show the power consumption for the ‘On’ and ‘Off’ states (Figure 12 and Figure 13), visually representing the advantages of the manual threshold method in aligning with the actual data distribution. A related study [64] developed a Kalman filter-based algorithm to detect sensor faults in WSN for irrigation, using predefined thresholds to isolate sensor faults. This showed the importance of accurate thresholding in signal classification systems.

However, a key limitation of the Min–Max threshold approach was the overlap of power values between the ‘On’ and ‘Off’ states, contributing to classification errors, especially false negatives. This reduced the accuracy of detecting the pump and valve states. Although the manual threshold method offered improved performance, it relied on subjective, manual observations to determine thresholds, which could be time-consuming and less scalable. This study presented a robust comparison between the Min–Max and manual threshold methods, highlighting the strengths of the manual approach in improving system performance while acknowledging its limitations.

The detection model demonstrated high performance across several key metrics, with the pump achieving an accuracy of 99.70% and both solenoid valves exceeding 98% accuracy, as shown in Table 5. This high level of reliability indicates the effectiveness of the model in distinguishing between normal and abnormal operations. Moreover, the precision for all components reached 100%, highlighting the system’s ability to detect abnormal conditions without false positives, a key factor for maintaining operational integrity. However, the system recorded several outliers, particularly for the pump (64 outliers) and the first solenoid valve (21 outliers). These outliers were primarily caused by irregularities in signal processing immediately following actuator state changes, introducing distortions in the time-series data. Adjusting the threshold range to more closely align the ‘On’ and ‘Off’ states could help mitigate these outliers and improve the precision in distinguishing between operational states without introducing additional noise. While the system showed high accuracy and precision, especially for solenoid valves, the presence of outliers and occasional inconsistencies in abnormal detection, particularly for the pump, indicate areas for further optimization. Refining the thresholds and incorporating more advanced detection algorithms could enhance the overall robustness of the irrigation system.

The comparison of fault detection models for sensors and actuator systems highlights the diverse approaches and techniques used in this domain, as summarized in Table 6. Signal processing and machine learning methodologies are the primary approaches for fault detection, utilizing system parameters such as soil temperature sensors, soil moisture content sensors, leaf-turgor pressure sensors, and voltage and current sensors to achieve varying levels of accuracy. The results indicate that the proposed method achieved competitive accuracy (99.2%) using power consumption analysis and signal processing. In comparison, similar studies by [28] utilized signal processing to detect valve failures using voltage and current signals, achieving an impressive accuracy of 98%. In contrast, several machine learning models were explored, including SVM-based and k-NN-based classifiers [27,64] and an IoT-based expert system [30]. These models achieve varying levels of accuracy between 84–99%. The comparison between different studies highlights the potential of signal processing-based approaches for effective fault detection in sensor and actuator systems. This study specifically focused on the real-time implementation of signal processing techniques to identify abnormalities in irrigation systems, aiming to enhance the reliability and efficiency of fault detection in such applications.

The technical results demonstrate high accuracy and reliability, with significant agricultural benefits. For example, reducing false negatives through manual thresholding minimizes instances of over- or under-irrigation. Additionally, the anomaly detection model effectively identifies irregularities, ensuring even water distribution and promoting healthy crop growth. Future research should focus on improving detection accuracy by integrating additional environmental sensor data and advanced machine learning techniques with signal processing to enhance anomaly classification and detection capabilities across diverse environmental and operational conditions. Additionally, evaluating the scalability and adaptability of the system for different types of actuators, along with the potential for real-time monitoring and integration with IoT frameworks, could further enhance its applicability in diverse agricultural settings, solidifying its role in precision agriculture.

## 5. Conclusions

This study aimed to develop an advanced ICT-based automated irrigation control and data acquisition system for agricultural applications. By employing relay modules controlled by a secondary MCU, the system automated irrigation processes while integrating sensors to monitor key environmental parameters, such as the soil water content, water potential, and leaf temperature. The integration of multiple MCUs, with data collected at 10-s intervals, ensured precise control and timely responses to environmental changes, contributing to optimized water usage. The developed smartphone application enabled real-time data reception and irrigation control, providing an effective tool for optimizing water management. The Python-based automation of the secondary MCU, coupled with multi-threading techniques, ensured seamless data management and prevented data loss during transmission using the MQTT protocol, with data stored as CSV files in the smartphone, facilitating further analysis.

Signal processing techniques, including the application of a first-order low-pass digital filter, significantly improved the accuracy of detecting operational states of the irrigation actuators by minimizing noise and suppressing sudden amplitude changes. Analysis of power consumption patterns, combined with histogram evaluations and threshold determination, allowed accurate classification of actuator states. The detection model showed better performance, with high accuracy, precision, recall, and F1 scores and an mAP of 99.76% for the second solenoid valve. Despite its high performance, inconsistency in recall indicated areas for further improvement. Future work should focus on refining the threshold settings and enhancing detection algorithms to further improve the system’s robustness. Overall, the developed system demonstrates good potential for reliable, real-time irrigation management and holds promise for broader applications in automated agricultural systems requiring precise control and early anomaly detection.

## Figures and Tables

**Table 1:** Specifications of the pump and solenoid valve were used in this study.

Water Pump	
Company	Shurflo
Model	8090-212-246
Check valve	(2-way operational) prevents reverse flow
Discharge rate	4.5 L/min
Pressure switch	Factory set @ 413.685 kPa, turn on 310.26 kPa ± 34.47 kPa
Prime	Self-priming up to 2.13 m vertical
Operating voltage	230 V AC
Average current	0.35 A
**Solenoid Valve**	
Company	Bermad
Model	S390-2W24VAC-R
Control type	2-way solenoid actuator
Average power	1.7 W
Average amps	0.125 A
Coil resistance	37.5 Ω

**Table 2:** Specifications of the current sensors used in this study.

Item	Specifications	
Model	ACS712ELC-05	ACS712ELC-20
Sensitivity	185 mV/Amp	185 mV/Amp
Operating voltage	5 V DC	5 V DC
Measuring range	−5 A to +5 A	−20 A to +20 A
Operating temperature	−40 °C to 85 °C	−40 °C to 85 °C
Noise	21 mV	11 mV

**Table 3:** Specifications of primary and secondary MCUs used in this study.

Primary Microcontroller		Secondary Microcontroller	
Model	Arduino Nano	Model	Raspberry Pi 4B board
CPU	ATmega328P	CPU	Quad-core Cortex-A72, 64-bit, 1.8 GHz
SRAM	2 KB	SRAM	8 GB LPDDR4-3200
Connection	22 GPIO pins (8 analog inputs, 14 digital IO pins)	Connection	standard 40-pin GPIO header
Operating system	None	Operating system	Linux based
Operating voltage	5 V DC	Operating voltage	5 V DC
Operating temperature	−40 °C to 85 °C	Operating temperature	00 to 500

**Table 4:** Summary of the abnormal actuator control operations identified by the detection system.

	Pump	Solenoid Valve 1	Solenoid Valve 2
Actual normal operation	387	387	333
Actual abnormal operation	6	6	60
Detected normal operation	322	359	316
Detected abnormal operation	0	0	58
Outlier	64	21	14

**Table 5:** Abnormal operation detection results with different performance indices.

	Accuracy	Precision	Recall	Specificity	F1 Score
Pump	99.70%	100%	99.69%	100%	99.84%
Solenoid valve 1	98.12%	100%	98.09%	100%	98.91%
Solenoid valve 2	98.68%	100%	98.44%	100%	99.22%

**Table 6:** Comparison of different fault detection models used for sensors and actuator fault detection using signal processing and machine learning models.

Model	Approach	Parameter	Accuracy (%)	Reference
Autoregressive model+ Kalman filter	Signal processing	Soil temperature, soil moisture content sensor values	-	[64]
Solenoid valve fault diagnosis (SVM model)	Machine learning	Time-frequency analysis, current signal	84–92	[27]
IoT-based expert system (SVM classifier	Machine learning	Leaf-turgor pressure sensor value	84.9	[30]
IoT-based expert system (kNN classifier)			84.8	
Auto-encoder-based fault diagnosis	Signal processing	Voltage signals, current signals	98	[28]
WSN fault detection system (SVM classifier)	Machine learning	Faulty sensor detection	99	[64]
Power level detection	Signal processing	Current sensor data	99.2	This study

## Data Availability

Data are contained within the article.
